# An Unusual Case of Sublingual Epidermoid Cyst Mimicking Plunging Ranula

**DOI:** 10.7759/cureus.42874

**Published:** 2023-08-02

**Authors:** Muhammad Asad Ullah, Awais Ahmed, Syed Muhammad Shahnawaz Hyder, Khalid Javed, Muhammad Qasim Naeem

**Affiliations:** 1 Radiology, Shaukat Khanum Memorial Cancer Hospital and Research Centre, Lahore, PAK; 2 Radiology, The Indus Hospital, Karachi, PAK

**Keywords:** intraoral, epidermoid inclusion cyst, giant epidermoid cyst, plunging ranula, sublingual

## Abstract

Epidermoid cyst in the oral cavity is uncommon. It is even more rare to see an epidermoid cyst in the sublingual region. We report the case of a 30-year-old male presenting with a swelling in the floor of the mouth extending into the submental and submandibular regions. The midline swelling was painless, soft, and dome-shaped. CT scan contrast revealed the site and extent of swelling. The complete surgical excision of the lesion was performed via a transcervical approach. Histopathology revealed cystic fibrocollagenous tissue covered by squamous epithelium containing some keratin flakes.

## Introduction

Epidermoid cysts are benign lesions, arising from the growth of epidermal cells in a confined area of the dermis [1]. They may develop anywhere in the body, most commonly involving the ovaries, testis, and sacrum. However, they may occur in the head and neck regions in 7% of cases, with very few occurring intraorally (1.6%) [2]. Only 0.01% of all cases are found sublingually in the floor of the mouth [3]. Though usually congenital, they often clinically present in the second or third decades of life [2].

Epidermoid, dermoid, and teratoid are the three types of squamous epithelium-lined cystic malformations, according to Meyers classification, with dermoid cyst often used as a broader term encompassing all of these. Etiologically, they may develop due to trauma and dysodontogenesis and from thyroglossal cyst [3].

In the floor of the mouth, they may occur above or below the mylohyoid muscle presenting as an asymptomatic painless midline swelling with slow but progressive growth [4]. When the cysts enlarge in the oral cavity, they may push the tongue posteriorly and interfere with articulation; mastication; swallowing; and, rarely, respiration. On extension into the submental space, they present as “double chin” [2].

## Case presentation

**Figure 1:**
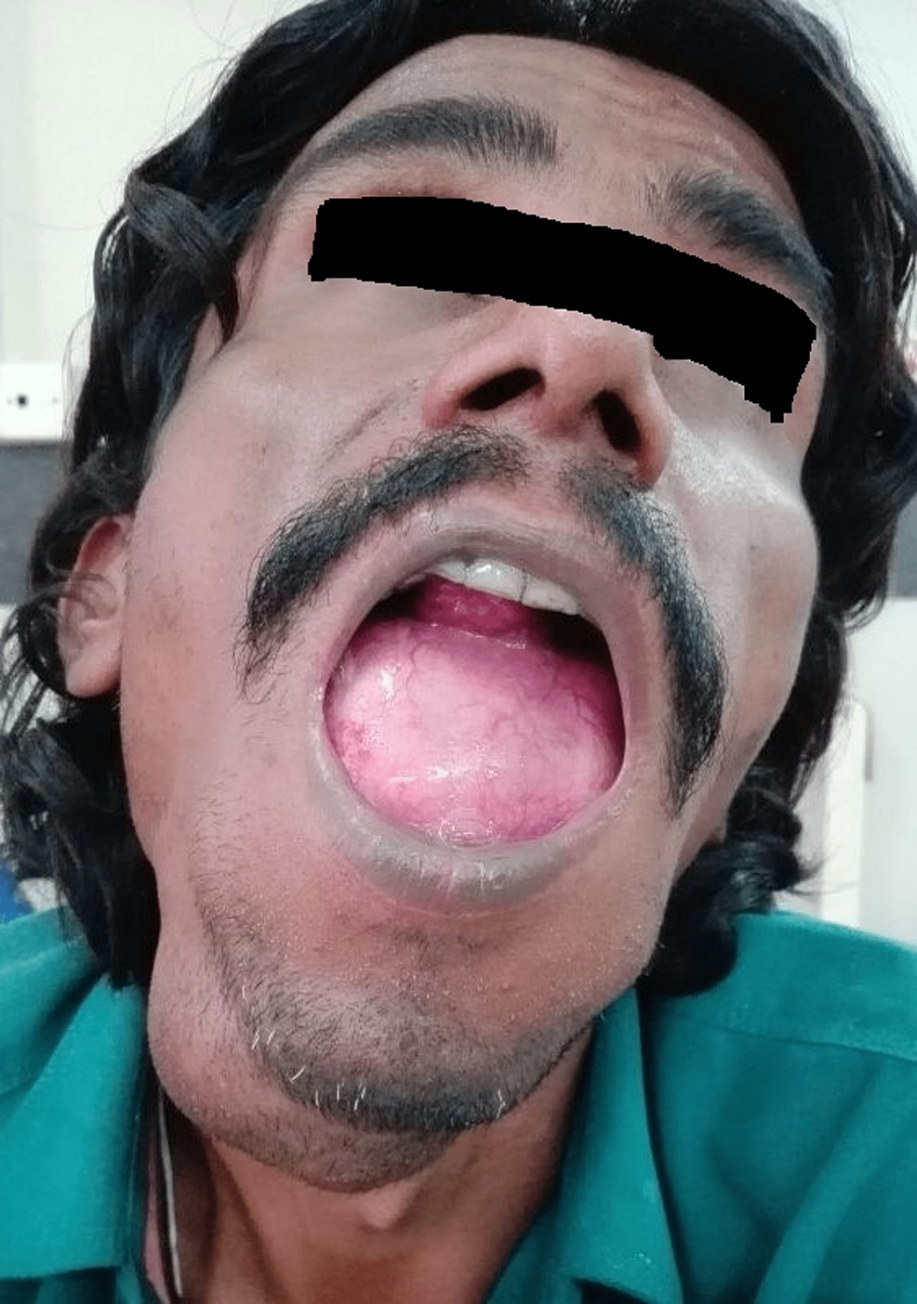
Large sublingual epidermoid cyst extending into the submental and submandibular regions

A 30-year-old male came to our department with the complaint of a painless swelling in the floor of the mouth, bulging into the submandibular region (Figure 1). Over many years, the sublingual lesion grew gradually. However, it becomes enlarged in size mainly over the course of the last three years; as such, it started to interfere with speech, swallowing, and mastication. Thankfully, there was no hindrance to respiration. It spontaneously ruptured a few years back, exuding around 500 mL of a grayish fluid with white granules. There was no notable medical history or background of trauma.

When the oral cavity was examined, a reddish dome-shaped enlargement was seen covering the entire gingivo-lingual sulcus and extending up to the anterior faucial pillars. It was soft, fluctuant, and compressible but non-reducible. Moreover, it was non-tender and non-pulsating.

Upon the examination of the neck, there was an inverted dome-shaped 13 × 11 cm swelling in the submental and submandibular regions extending from the angle of the mandible to the hyoid bone inferiorly. There was no discoloration of the overlying skin. Transillumination was absent. There was no enlarged cervical lymph node palpated.

**Figure 2:**
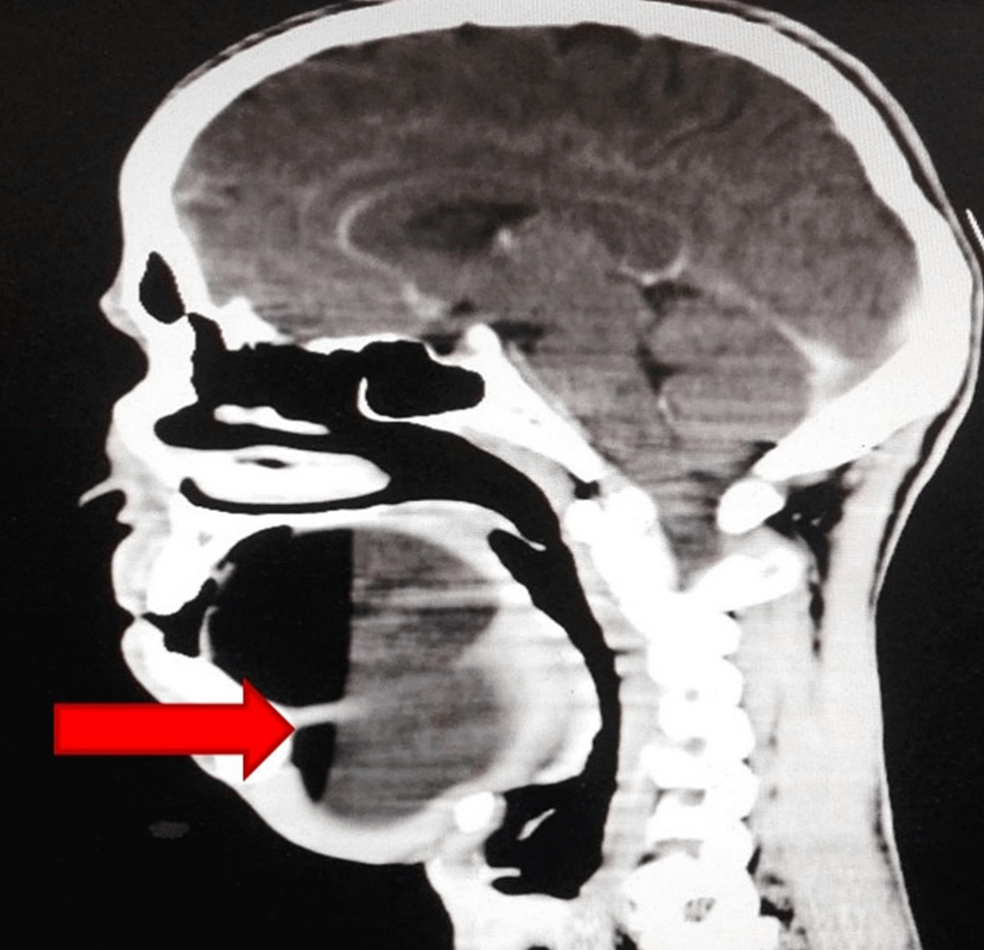
CT scan sagittal view showing non-enhancing mass in the floor of mouth

A CT scan of the head and neck with contrast was performed. It showed a large mass of 10 × 7 × 5 cm with a fat-fluid level, extending from the soft palate to the floor of the mouth and submental region (Figure 2). It was abutting the hard palate and the mandible anteriorly while pushing the tongue posteriorly. There was no cervical lymphadenopathy.

**Figure 3:**
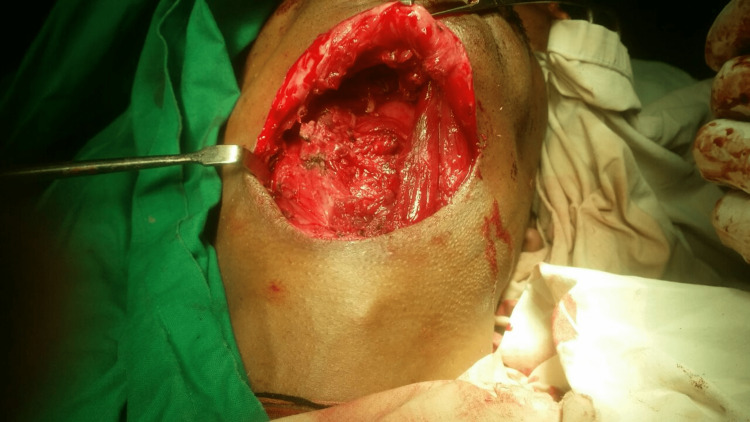
Perioperative picture showing the transcervical approach for the removal the large epidermoid cyst

The surgical removal of the lesion was planned. General anesthesia was given following intranasal fiber-optic intubation (due to the obstructing oral cavity lesion). The lesion was approached via the neck (Figure 3) through a midline skin crease incision and was exposed by subplatysmal dissection. By careful dissection, the thin wall of the cyst was separated from the surrounding tissues and was excised in toto. The sublingual glands were also removed.

**Figure 4:**
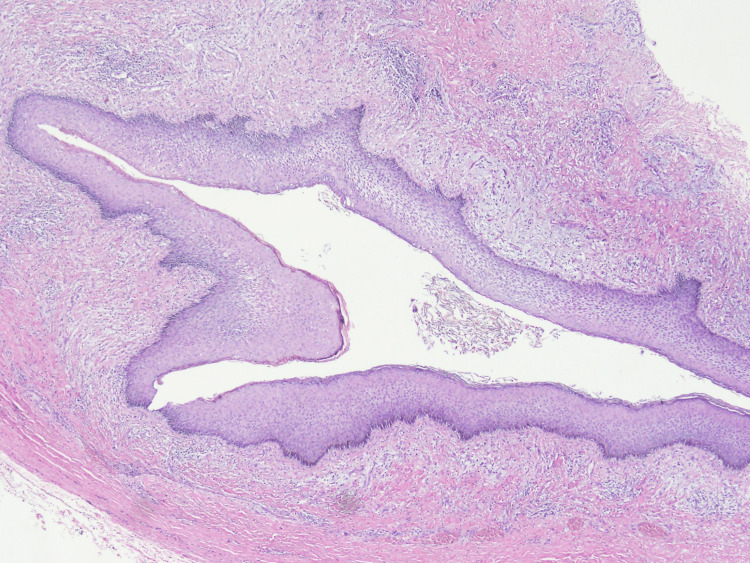
H&E-stained section showing a cyst lined by stratified squamous epithelium without any associated skin adnexal structures with underlying fibromuscular tissue showing inflammatory cells The luminal side of the cyst shows keratin flakes

The excised specimen was sent for histopathological evaluation, which revealed cystic fibrocollagenous tissue focally covered by squamous epithelium. The cyst showed a denuded lining and some keratin flakes (Figure 4). The tissue showed fibrosis, congested vessels, and mild inflammation. Focal salivary gland parenchyma was also seen.

## Discussion

Epidermoid cysts are nonmalignant inclusion cysts lined by the ectoderm alone [5]. Etiologically, they may be dysontogenetic, originating from the entrapment of the ectoderm during the fusion of the first and second branchial arches in the third and fourth weeks of embryonic life. They may also develop later in life as an acquired lesion, secondary to some trauma resulting in the embedment of epithelial cells into the deeper tissues. Another theory suggests thyroglossal anomaly as a cause [2,6]. The epidermoid cyst usually presents in the second and third decades of life. It is rare in childhood. The lesion has no sex predilection, affecting both genders equally [6,7]. Epidermoid and dermoid cysts occurring in the head and neck constitute only about 7% of these cysts. They are uncommon intraorally (1.6%) with only 0.01% of cases reported on the floor of the mouth [2,8].

Histologically, according to Meyers classification, these squamous epithelium-lined cystic malformations are classified as epidermoid, dermoid, and teratoid. They all contain keratin and sebaceous fluid. However, epidermoid cysts are lined by only epidermal-type squamous epithelium, while dermoid cysts in addition include skin appendages, and teratoid cysts have their lining derived from all three germinal layers [9]. Anatomically, cysts in the floor of the mouth can be categorized into three types, sublingual, submental, and submandibular cysts, depending upon their relationship with the mylohyoid, geniohyoid, and genioglossus muscles [2,6].

Clinically, epidermoid cysts are usually painless and asymptomatic. However, larger ones may push the tongue backward interfering with mastication, articulation, swallowing, and sometimes respiration. Moreover, they are well-encapsulated, slow-growing cysts and soft to doughy in consistency and usually present without lymph node involvement [5-7].

The differential diagnoses of epidermoid cyst can broadly be classified as infective (cellulitis of the floor of the mouth, necrotizing lymphadenitis, and abscess formation), neoplastic (salivary gland tumors), developmental (branchial cleft cyst, cystic hygroma, and thyroglossal cyst), and anatomic abnormalities (lymphangioma, mucocele, hemangioma, lipoma, and neurofibroma) and mucous extravasation (ranula). The absence of inflammatory signs helped us exclude infectious lesions. While the benign appearance and the lack of lymph node involvement reduced the likelihood of malignancy [7-11]. Since the lesion in our case had a prominent oral component along with submental and submandibular swelling, we ruled out developmental and anatomic abnormalities of the neck. Hence, we were left with the diagnostic possibility of an epidermoid cyst and ranula. Plunging ranula topped our differential list because of the clinical appearance of the lesion and also because it is more common.

Imaging methods are useful in establishing the diagnosis alongside clinical examination. Despite the fact that MRI, CT, and US are of immense utility in diagnosing the lesion and surgical planning, we can only shortlist differentials on the basis of imaging. Fine-needle aspiration cytology (FNAC) also has a low sensitivity and specificity for the diagnosis of these lesions. Hence, histopathology is the only effective method for accurately diagnosing the disease [4].

The complete surgical excision of the cyst is the recommended treatment in these cases to validate the diagnosis and relieve the patient from difficulty in mastication, articulation, and swallowing. Any obstruction to the laryngeal airway due to the posterior bulge of the tongue may lead to grievous consequences; hence, surgical excision is necessary. Alternatively, marsupialization with intralesional packing can be done [9]. The exact site of the lesion determines the surgical approach. Small cysts above the mylohyoid muscle are removed by giving intraoral incisions, while those below the mylohyoid are best approached extraorally via submental or submandibular incision [4,9]. The malignant transformation of the lesion is rare. After the adequate removal of the lesion, the prognosis is good with a very low incidence of relapses [1,9].

## Conclusions

Although intraoral cystic lesions in the floor of the mouth are rare, epidermoid inclusion cyst should be considered in the differential diagnosis. The treatment of epidermoid cysts in the floor of the mouth is surgical and can be intraoral or extraoral according to the localization and the size of the lesion.
